# Controlling Nutritional Status Score as a Predictor for Chronic Obstructive Pulmonary Disease Exacerbation Risk in Elderly Patients

**DOI:** 10.3390/metabo13111123

**Published:** 2023-11-02

**Authors:** Aurelio Lo Buglio, Giulia Scioscia, Francesco Bellanti, Pasquale Tondo, Piera Soccio, Matteo Pio Natale, Donato Lacedonia, Gianluigi Vendemiale

**Affiliations:** 1 Department of Medical and Surgical Sciences, Institute of Internal Medicine, University of Foggia, Policlinico of Foggia, 71122 Foggia, Italy; francesco.bellanti@unifg.it (F.B.); gianluigi.vendemiale@unifg.it (G.V.); 2 Department of Medical and Surgical Sciences, Institute of Respiratoy Diseases, University of Foggia, Policlinico of Foggia, 71122 Foggia, Italy; giulia.scioscia@unifg.it (G.S.); pasquale.tondo@unifg.it (P.T.); piera.soccio@unifg.it (P.S.); matteo.natale@unifg.it (M.P.N.); donato.lacedonia@unifg.it (D.L.)

**Keywords:** malnutrition, COPD, AECOPD, elderly, CONUT score

## Abstract

The Controlling Nutritional Status (CONUT) score is a simple screening tool able to assess poor nutritional status as well as to predict clinical adverse outcomes in different clinical settings. No data are available in older patients with chronic obstructive pulmonary disease (COPD). This study aimed to investigate the CONUT score as a predictor of frequent exacerbations. We retrospectively enrolled 222 patients aged 65 years or older, classified in two groups according to the number of exacerbations (or hospitalizations because AECOPD) during the previous year. The two groups were further divided according to low (<5) or high (≥5) CONUT scores. A total of 67.2% of frequent exacerbators had a high CONUT score. These patients exhibited a significantly higher CAT score, lower FEV1 percentage value, and higher prevalence of severe GOLD stages compared to those with low CONUT. Multivariate analysis showed that a CONUT score ≥ 5 was the best independent predictor (OR 20.740, *p* < 0.001) of the occurrence of ≥2 exacerbations (or 1 hospitalization) during the previous year. The CONUT score seemed to have a high prognostic value for frequent exacerbations for COPD in older patients. The predictive role of different CONUT score cut-off values needs to be validated in larger COPD populations in future multi-center, prospective clinical studies.

## 1. Introduction

Chronic obstructive pulmonary disease (COPD) is a heterogeneous lung condition characterized by chronic respiratory symptoms (dyspnea, cough, expectoration) due to airway abnormalities causing a persistent and progressive airflow obstruction [1]. The progression of the disease results in the occurrence of acute exacerbation of chronic obstructive pulmonary disease (AECOPD) that is characterized by a worsening of symptoms, including cough, increased sputum, wheezing, and occasionally fever and widespread pain [2].

AECOPD leads to an increased need for hospitalization and an increased risk of death in patients with COPD [2]. Additionally, AECOPD is responsible for a large proportion of the healthcare costs attributable to COPD. The occurrence of AECOPD is worsened by the increase in susceptibility to respiratory infections of viral, bacterial, or fungal origins, as well as exposure to air pollution or common allergens [3].

To date, the onset of AECOPD is still unclear and, due to the heterogeneity of the disease, some patients with COPD have frequent AECOPD (FE, frequent exacerbation), while others rarely suffer from AECOPD (IE, infrequent exacerbation) [2].

COPD is associated with multiple systemic manifestations, including impaired nutritional status or malnutrition, especially in elderly patients [4,5]. A poor prognosis is often associated with an altered nutritional status. Therefore, assessing the nutritional status of the patient in many diseases is crucial to defining the short-term prognosis and the risk of mortality as well as taking corrective action [6,7]. Data from other studies indicate that 30%–60% of the patients hospitalized with COPD have an impaired nutritional status, depending on the different diagnostic methods and criteria used, and this condition has a negative impact on prognosis, including a higher risk of hospitalization, poor exercise tolerance, severe airflow obstruction, or mortality [8]. Although a wide range of therapeutic approaches can be used, malnutrition remains underdiagnosed and undertreated in COPD patients.

The Controlling Nutritional Status (CONUT) score is a recent nutritional marker based on the values of serum albumin, the absolute number of lymphocytes, and the value of serum of cholesterol [9]. It is based on a simple calculation from data collected by routine biochemical analysis and complete blood count that allows for assessing the nutritional status of the patient [9]. The CONUT score has a high prognostic value in specific populations such as the elderly, cancer patients, gastroenterological, or patients affected by heart failure or ischemic stroke [10,11]. In elderly patients, the CONUT score has demonstrated a high predictive value for a longer length of stay (LOS) and a higher risk of in-hospital mortality without incurring additional costs because the parameters used are often included in routine lab tests performed upon patient admission [6,11].

The predictive role of the CONUT score in elderly outpatients with COPD has not been established yet. This study aimed to assess the utility of the CONUT score in non-hospitalized elderly patients with COPD to identify individuals at higher risk of exacerbation and to investigate whether there is an association between the score and the severity of COPD-related symptoms.

## 2. Materials and Methods

### 2.1. Study Population and Design

Sixty-five years or older patients with a diagnosis of COPD were retrospectively enrolled through the outpatient clinic of the Institute of Respiratory Diseases, Policlinico of Foggia, Italy, between January and July 2023.

COPD diagnosis was performed according to Global Initiative for Chronic Obstructive Lung Disease (GOLD) guidelines [1]. Patients were classified as FE or IE if they had equal or more than 2 exacerbations in the previous year (or 1 hospitalization) or equal or less than 1 exacerbation. The definition of AECOPD was based on the need for antibiotics and/or steroids treatment or hospitalization [1]. Exclusion criteria were as follows: patients with unstable medical conditions, respiratory failure, severe psychiatric illness, and/or patients that received treatment with sedatives or enrolled in other clinical trials.

**Figure 1:**
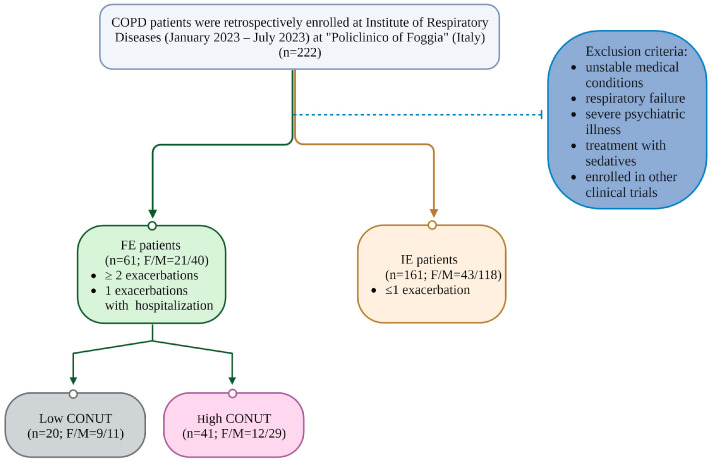
Flow chart of the study.

Patients were divided into two groups based on frequency of AEOCPD occurrence: FE if they reported ≥ 2 exacerbations (or 1 required hospitalization because of AEOCPD) during the previous year or IE if they had ≤1 exacerbation. FE patients were further assessed for the nutritional status. See Figure 1.

### 2.2. Data Collection

All subjects enrolled underwent collection of demographic and clinical data. Smoke habit, systemic and pulmonary comorbidities, Modified British Medical Research Council (mMRC) questionnaire score [12], and COPD Assessment Test (CAT) score [13], and the number of moderate and severe COPD exacerbations occurring during the previous year was also recorded [14]. Pulmonary function tests were performed. FEV1 and FVC were measured using a spirometer (Sensormedics, Milan, Italy). The best value of three maneuvers was expressed as a percentage of the predicted normal value. International standards were used to determine functional results in all patients [15,16].

### 2.3. Biochemical Analysis

Blood sampling was carried out on all patients to determine hemoglobin, white blood cells (WBCs), lymphocytes, glucose, albumin, total cholesterol, creatinine, triglycerides, and C-reactive protein (CRP).

### 2.4. Nutritional Assessment

Height and body weight were measured according to standardized procedures.

Body mass index (BMI) was calculated as the ratio between body weight and square height in meters. Nutritional status was evaluated using the CONUT score. CONUT score is calculated based on biochemical parameters: serum albumin (g/dL), total lymphocyte count (count/mm^3^), and total cholesterol (mg/dL), as reported in Table 1 [9]. FE patients were further divided in two subgroups—high CONUT and low CONUT, according to the presence of a CONUT score ≥5 or <5, respectively.

### 2.5. Statistical Analysis

Continuous variables were expressed as mean ± standard deviation of the mean (SD) or median (Interquartile Range, IR) and analyzed using Student’s *t*-test or Mann–Whitney’s test. Nominal and categorical variables were expressed as *n* (%) and analyzed using the Chi-Square test or Fisher’s exact test. Parametric or non-parametric distribution was evaluated using the Kolmogorov–Smirnov test.

We conducted univariate binary logistic regression analysis to examine the association between the FE phenotype and various factors, including age (continuous variable), gender (M/F), CRP (continuous variable), BMI (categorized as low if BMI < 25 kg/m^2^ and high if BMI ≥ 25 kg/m^2^), CONUT score (categorized as low if CONUT < 5 and high if CONUT ≥ 5), comorbidities (categorized as low if comorbidities < 3 and high if comorbidities ≥ 3), current smoking status, presence of bronchiectasis, and presence of emphysema. Variables found to be significant in the univariate analysis were included in a multivariate logistic regression model using the forced entry method to identify the risk factors significantly associated with the FE phenotype. *p* values < 0.05 were considered statistically significant. Statistical analysis was performed with the Statistical Package for Social Sciences version 23.0 (SPSS, Inc., Chicago, IL, USA) and the package Graph-Pad Prism 6.0 for Windows (GraphPad Software, Inc., San Diego, CA, USA).

## 3. Results

### 3.1. Baseline Characteristics of Patients

A total of 222 COPD patients were enrolled in the study, including 64 females (28.8%) with an average age of 71.9 ± 5.3 years. Patients were divided in two groups based on exacerbations or required hospitalizations during the previous year: 161 (72.5%) were included in the COPD IE group (COPD class A 98 (44.1%) and class B 63 (28.4%)) and 61 (27.5%) in the COPD FE group (COPD class E). Baseline characteristics are summarized in Table 2.

No significant differences were found between FE and IE regarding sex, serum levels of hemoglobin, platelets, glucose, creatinine, and total cholesterol. FE were older (*p* < 0.001) with lower values of lymphocytes (*p* < 0.001), albumin (*p* < 0.005), and BMI (*p* < 0.001). Conversely, they presented significantly higher WBC (*p* < 0.001), eosinophil (*p* < 0.001), and CRP (*p* < 0.001) as compared to IF (Table 2). The FE group exhibited a higher prevalence of comorbidities (*p* < 0.026) and bronchiectasis (*p* < 0.013) than the IF group, with no significant differences in the prevalence of current smokers and emphysema between the two groups. As expected, the FE group showed a more pronounced overall impact of COPD on the quality of life, as assessed by the CAT score (*p* < 0.001), in comparison to the IF group, along with a higher level of dyspnea, as evaluated by the mMRC score (*p* < 0.001). Remarkably, significantly higher mean values of CONUT score were found in the FE group than in the IE group (*p* < 0.001). According to the frequent exacerbation phenotype, in this group, lower median FEV1 percentages were found with respect to patients with an infrequent exacerbation phenotype (*p* < 0.001) (Table 2).

### 3.2. Patients’ Characteristics According to CONUT Score in the Frequent Exacerbation Group

**Figure 2:**
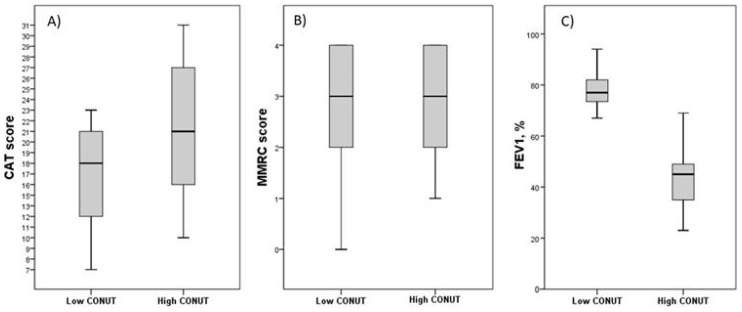
Differences in symptoms and respiratory performances in FE groups according to CONUT score. Data are reported as median and IQR values. Abbreviation: CONUT, controlling nutritional status; CAT, COPD assessment test; mMRC, Modified Medical Research Council; FEV1, Forced Expiratory Volume in the first second; IQR, interquartile range. (**A**) Differences in CAT score between groups according to CONUT score. *p* = 0.019; (**B**) Differences in mMRC score between groups according to CONUT score. *p* = 0.844; (**C**) Differences in FEV1 percentage value between groups according to CONUT score. *p* < 0.001. Statistical differences were assessed using Mann–Whitney U Test.

**Figure 3:**
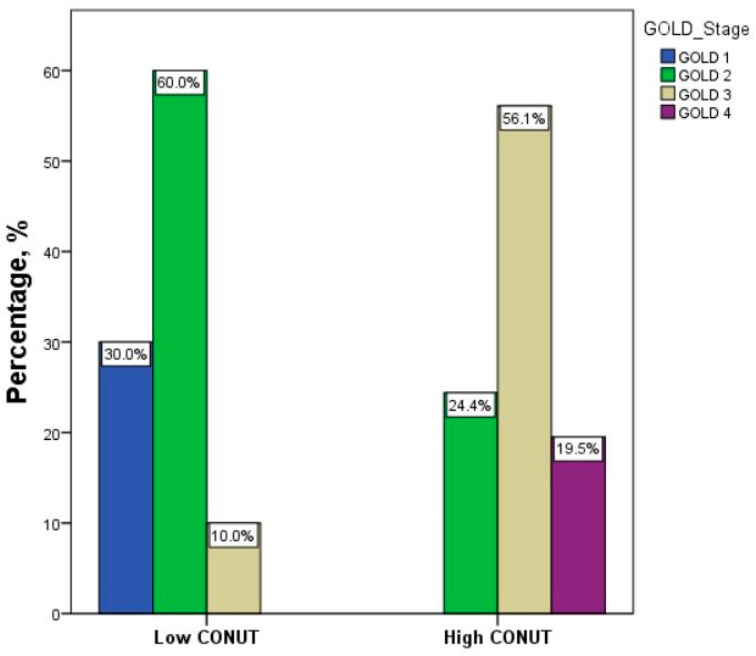
Distribution of severity of GOLD stages between groups according to CONUT score. Abbreviation: CONUT, controlling nutritional status. Statistical differences were assessed using Fisher’s exact test. *p* < 0.001.

Table 3 summarizes the data of patients with FE stratified according to the CONUT score. Forty-one patients (67.2%) had a high CONUT score. The group with a high CONUT score exhibited lower lymphocyte counts, total cholesterol levels, and albumin levels compared to the group with a low CONUT score (*p* < 0.001). This variation depends on the utilization of CONUT scores for patient categorization. No significant differences were observed in terms of age, sex, hemoglobin levels, white blood cell count, eosinophil count, platelet count, glucose levels, creatinine levels, and C-reactive protein (CRP) serum concentrations between the two groups. Similarly, there were no discernible variations in BMI, prevalence of comorbidities, number of current smokers, presence of emphysema, or bronchiectasis between the two groups. Interestingly, the group with a high CONUT score exhibited significantly higher median CAT scores (*p* < 0.001) than the group with a low CONUT score. However, when we analyzed the median values of mMRC, the two groups did not show significant differences (Table 3 and Figure 2A,B). Furthermore, patients with a high CONUT score exhibited lower FEV1 percentage values compared to patients with a low CONUT score (Table 3 and Figure 2C). GOLD stages differed significantly between groups, with a distribution of severity of GOLD grades tending to shift toward more severe grades in patients with a high CONUT score: patients with a low CONUT score presented a major prevalence of the GOLD 2 stage (60%), followed by GOLD 1 (30%) and GOLD 3 (10%); among patients with a high CONUT score, it was the GOLD 3 stage (56.1%), followed by GOLD 2 (24.4%) and GOLD 4 (19.5%) (*p* < 0.001) (Table 3 and Figure 3).

### 3.3. Multivariate Analysis for the Association of Risk Factors with AECOPD (Acute Exacerbations of Chronic Obstructive Pulmonary Disease)

**Figure 4:**
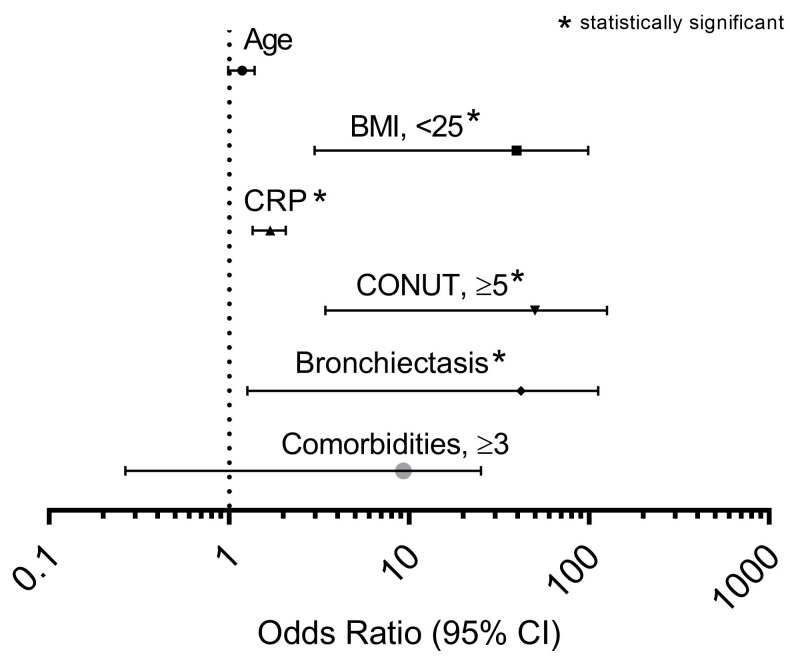
Odds ratios of factors associated with ≥2 exacerbations (or required 1 hospitalization because AOCPD) during previous year in a multivariate logistic regression model applied in the entire cohort studied. Abbreviation: BMI, body mass index; CRP, C reactive protein; CONUT, controlling nutritional status. The asterisk identifies the independent variables that have been found statistically significant.

A multivariate analysis was performed to verify the most important factors associated with the occurrence of ≥2 exacerbations (or required 1 hospitalization because AECOPD) during the previous year, showing that CONUT score ≥ 5 was the best independent predictor (OR 20.740, *p* < 0.001), followed by the presence of a BMI < 25 kg/m^2^ (OR 17.154, *p* = 0.001), bronchiectasis (OR 11.886, *p* = 0.031), and plasma CRP levels (OR 1.668, *p* < 0.001), as shown in (Figure 4).

## 4. Discussion

This study examined the CONUT score as a predictor tool of frequent exacerbations in elderly outpatients with COPD. The principal findings were: (1) high CONUT score is the stronger independent predictor of frequent exacerbators; (2) high CONUT score is associated with more severe symptoms and lower FEV1 percentage values among FE patients.

COPD is a prevalent disease and a leading cause of morbidity and mortality worldwide [4]. AECOPD represents a major concern in the clinical management of patients with COPD because often leads to hospitalization, clinical death, and the worsening of economic burden in these patients [17,18]. In the population studied, we found a prevalence of FE of 27.5% according to the Evaluation of COPD Longitudinally to Identify Predictive Surrogate End-points (ECLIPSE) study, where the FE phenotype was identified in 22 to 47% of patients depending on GOLD stages [19]. In our study population, patients in the FE group were older than the comparison group. In fact, previous data showed a greater risk of exacerbation in older COPD patients than in younger ones, with a 20% increase in risk for every 10 years of age [20]. The increased experience of AECOPD with age is probably due to biological and environmental factors [21]. The higher CAT scores and lower FEV1 values in the frequent exacerbator group, when compared to the infrequent exacerbator group, confirm the greater likelihood of frequent exacerbators to experience a less favourable clinical course, marked by a more rapid decline in lung function and poorer clinical outcomes [22]. Also, the inflammatory status plays a significant role in the evolution of COPD, and there is strong associative evidence that the inflammatory processes in COPD increase the risk of poor clinical outcomes, such as cardiovascular disease (CVD) and lung cancer [23,24]. Enhancing our comprehension of FE phenotype and investigating a novel biomarker for improved diagnosis are imperative steps in the development of precision medicine strategies [22]. Strong links have been demonstrated to exist between malnutrition and inflammation to the extent that inflammation is one of the criteria used in the diagnosis of malnutrition according to the Global Leadership Initiative on Malnutrition (GLIM) [25,26]. In this scenario, malnutrition plays a key role in COPD patients by reducing muscle mass and decreasing the strength and endurance of respiratory muscles. Furthermore, malnutrition is associated with the severity of the disease and is related to increased exacerbations, hospitalization, and prolonged hospital stays [27].

The most important result of our study is the evidence of a strong association between the CONUT score ≥ 5 with the FE phenotype. Specifically, in the multivariate analysis, a high CONUT score is the strongest predictor of frequent exacerbations independent of other risk factors such as age, gender, bronchiectasis, or inflammation. In fact, we found a higher CONUT score in the FE group than in the IF group. The CONUT score is a simple and reliable screening tool to identify patients with poor nutritional status in both inpatient and outpatient settings [6,28]. There are limited data in the literature regarding the association between the CONUT score and COPD. In the FE group, we found that more than 67% of patients had a high CONUT score, with no significant differences in terms of age, sex, comorbidities, and other risk factors such as current smokers, the prevalence of emphysema, or bronchiectasis. Additionally, there were no differences in inflammatory status based on the CONUT score. However, FE patients with a high CONUT score showed a higher CAT score and FEV1 percentage value. The CAT is a short, self-administered quality-of-life questionnaire that provides a good sense of the health impact in COPD patients. Also, the CAT score is related to the severity of exacerbation and predicts recurrence [29]. García-Sidro et al., reported that in hospitalized patients, the CAT score predicts new exacerbation, readmission, or death in the subsequent three months when there is a change of ≤4 points in the CAT score at discharge compared to that obtained at admission [30]. In our study, a high CONUT score identified the patients with a more severe impact of COPD on a life. However, no differences were found in the median value of mMRC. FEV1 is strongly related to mortality in a general population, although there are conflicting data [31]. Recently, the role of FEV1 as a predictor for all causes of mortality was confirmed [31]. We observed a lower FEV1 percentage value among FE patients with a CONUT score of ≥5 as well as a higher prevalences of GOLD stages C and D, suggesting a potential higher risk for this subgroup of patients in terms of clinical outcomes. However, it is important to note that this association requires further confirmation.

To the best of our knowledge, this is the first study to investigate the prognostic value of the CONUT score in AECOPD outpatients. However, several limitations should be highlighted. First, this is a retrospective, single-center study, so the results may not be generalized to other populations and may be influenced by potential confounding factors. Second, the small sample size restricted the subgroup analysis, and the heterogenic distribution of the sample size could have affected the statistical power of the study. The limitations associated with a single-center study and a relatively small sample size can potentially be addressed through future research endeavors, particularly by conducting multicenter studies. Expanding the sample size would help mitigate these limitations, leading to more generalizable results applicable to a broader population. Third, data on statin use were unavailable. We must consider that statin use can affect the CONUT score by influencing cholesterol levels. Finally, we conducted a comprehensive statistical adjustment for the measured confounders. However, it is important to note that there are unmeasured variables that can affect the risk of exacerbation, including the medications administered and adherence to therapy.

Despite these limitations, our findings provide evidence of the potential role of the CONUT score in identifying older patients affected by COPD at a high risk of exacerbation. Considering the user-friendliness of this score and the common availability of the data to calculate it, the CONUT score could be routinely used in order to identify high-risk patients for exacerbation and allow for a more effective clinical management approach characterized by closer follow-up and more frequent therapy reassessments. Moreover, within the frequent exacerbator group, a high CONUT score identifies a subset of patients with severe disease manifestations, facilitating the implementation of preventive and therapeutic measures for this specific patient subgroup.

Further specific validation studies are needed to confirm the reliability of CONUT in elderly patients with COPD for predicting both exacerbation risk and episode severity. These ongoing validations will help pave the way for future research, allowing us to explore potential applications and refine clinical guidelines.

## 5. Conclusions

In conclusion, the CONUT score shows high prognostic value for a FE phenotype in elderly outpatients affected by COPD as well as for identifying the patients with more severe symptoms. These data suggest a possible use of the CONUT score as a nutritional screening tool for identifying COPD patients at a higher risk of exacerbations and with a more severe impact on the quality of life. The predictive role of different CONUT score cut-off values needs to be validated in populations in future multi-center, large-sample, prospective clinical studies.

## Figures and Tables

**Table 1:** CONUT score calculation.

	Nutritional Status			
Variables	Normal	Light	Moderate	Severe
Albumin (g/dL) Score	≥3.5 0	3.0–3.49 2	2.5–2.9 4	<2.5 6
Total lymphocyte (n/mm^3^) Score	>1600 0	1200–1599 1	800–1199 2	<800 3
Total cholesterol (mg/dL) Score	>180 0	140–180 1	100–139 2	<100 3
Screening total score	0–1	2–4	5–8	9–12

Abbreviation: CONUT, controlling nutritional status.

**Table 2:** Baseline characteristics according to study groups.

	COPD IE *n* = 161 (72.5%)	COPD FE *n* = 61 (27.5%)	*p* Value
Age, years	70.9 ± 5.1	74.4 ± 5.0	**<0.001**
Sex F, *n* (%)	43 (26.7)	21 (34.4)	0.257
Hemoglobin, g/dL	14.2 ± 1.8	13.8 ± 1.7	0.463
WBC, n/mm^3^	7840 [6370 to 8670]	12,500 [9520 to 18,050]	**<0.001**
Lymphocytes, n/mm^3^	1656 [1287 to 2101]	1090 [919 to 1302]	**<0.001**
Eosinophils, n/mm^3^	112 [87 to 168]	340 [285 to 396]	**<0.001**
Platelets, ×10^9^/L	226 [189 to 256]	257 [205 to 321]	0.097
Glucose, mg/dL	121.5 ± 42.0	119.7 ± 30.6	0.890
Creatinine, mg/dL	0.84 [0.76 to 1.10]	0.86 [0.79 to 1.01]	0.889
Total cholesterol, mg/dL	164.1 ± 36.1	165.4 ± 34.0	0.906
Albumin, g/dL	3.9 ± 0.4	3.2 ± 0.6	**0.005**
CRP, ng/mL	5.7 [2.6 to 9.8]	22.1 [15.6 to 26.1]	**<0.001**
BMI, kg/m^2^	28.3 ± 4.2	24.0 ± 3.8	**<0.001**
Co-morbidities ≥ 3, *n* (%)	26 (16.1)	43 (29.5)	**0.026**
Current smokers, *n* (%)	68 (42.2)	21 (34.4)	0.289
Emphysema, *n* (%)	39 (24.2)	14 (23)	0.843
Bronchiectasis, *n* (%)	11 (6.8)	11 (18)	**0.013**
CONUT score	1 [0 to 1]	7 [4 to 8]	**<0.001**
CAT score	9 [7 to 14]	20 [13 to 24]	**<0.001**
mMRC score	2 [1 to 3]	3 [2 to 4]	**0.046**
FEV1, %	78 [63 to 92]	49 [39 to 73]	**<0.001**

Data are expressed as mean (±standard deviation), median [interquartile range], or *n* (percentage) as appropriate. Abbreviations: F, female; WBC, white blood cells; CRP, C reactive protein; BMI, body mass index; CONUT, controlling nutritional status; CAT, COPD assessment test; mMRC, Modified Medical Research Council; FEV1, Forced Expiratory Volume in the 1st second. *p* value < 0.05 was considered statistically significant (in bold).

**Table 3:** Clinical and biochemical characteristics according to CONUT score in patients with frequent exacerbations.

	Low CONUT *n* = 20 (32.8%)	High CONUT *n* = 41 (67.2%)	*p* Value
Age, years	74.3 ± 4.98.7	74.4 ± 5.1	0.920
Sex F, *n* (%)	9 (45.0)	12 (29.3)	0.260
Hemoglobin, g/dL	13.9 ± 0.6	13.8 ± 1.8	0.974
WBC, n/mm^3^	13300 [12,450 to 14,325]	9660 [7845 to 17,380]	0.190
Lymphocytes, n/mm^3^	1311 [1112 to 1880]	989 [721 to 1100]	**<0.001**
Eosinophils, n/mm^3^	337 [287 to 394]	348 [278 to 398]	0.731
Platelets, ×10^9^/L	276 [245 to 299]	238 [189 to 350]	0.269
Glucose, mg/dL	104.5 ± 17.7	122.1 ± 31.9	0.470
Creatinine, mg/dL	0.91 [0.88 to 0.96]	0.85 [0.79 to 1.03]	0.672
Total cholesterol, mg/dL	191.3 ± 23.9	139.5 ± 19.6	**<0.001**
Albumin, g/dL	3.7 ± 0.5	2.8 ± 0.4	**<0.001**
CRP, ng/mL	20.9 [13.3 to 28.9]	22.9 [17.3 to 25.7]	0.923
BMI, kg/m^2^	24.3 ± 4.3	23.9 ± 3.7	0.729
Co-morbidities ≥ 3, *n* (%)	4 (20.0)	14 (34.1)	0.372
Current smokers, *n* (%)	6 (30.0)	15 (36.6)	0.776
Emphysema, *n* (%)	4 (20.0)	10 (24.4)	0.704
Bronchiectasis, *n* (%)	5 (25.0)	6 (14.6)	0.479
CAT score	17 [11 to 21]	21 [15 to 27]	**0.019**
mMRC score	3 [2 to 4]	3 [2 to 4]	0.844
FEV1, %	77 [73 to 82]	45 [34 to 49]	**<0.001**
GOLD stage			
1, *n* (%)	6 (30)	0 (0)	
2, *n* (%)	12 (60)	10 (24.4)	**<0.001**
3, *n* (%)	2 (10)	23 (56.1)	
4, *n* (%)	0 (0)	8 (19.5)	

Data are expressed as mean (±standard deviation), median [interquartile range], or *n* (percentage) as appropriate. Abbreviation: CONUT, controlling nutritional status; F, female; WBC, white blood cells; CRP, C reactive protein; BMI, body mass index; CAT, COPD assessment test; mMRC, Modified Medical Research Council; FEV1, Forced Expiratory Volume in the first second. *p* value < 0.05 was considered statistically significant (in bold).

## Data Availability

The study data are available on reasonable request to the corresponding authors. The data are not publicly available due to privacy reasons.
